# An activating calcium-sensing receptor variant with biased signaling reveals a critical residue for Gα11 coupling

**DOI:** 10.1093/jbmr/zjae199

**Published:** 2024-12-10

**Authors:** Matthew R Benson, Rachael A Wyatt, Michael A Levine, Caroline M Gorvin

**Affiliations:** Division of Endocrinology, Diabetes & Metabolism, Nemours Children’s Health, Jacksonville, FL 32207, United States; Institute of Metabolism and Systems Research (IMSR) and Centre for Diabetes, Endocrinology and Metabolism (CEDAM), University of Birmingham, Birmingham, B15 2TT, United Kingdom; Centre for Membrane Proteins and Receptors (COMPARE), Universities of Birmingham and Nottingham, Birmingham, B15 2TT, United Kingdom; Division of Endocrinology and Diabetes, Department of Pediatrics, Center for Bone Health, The Children’s Hospital of Philadelphia, Perelman School of Medicine, University of Pennsylvania, Philadelphia, PA 19104, United States; Institute of Metabolism and Systems Research (IMSR) and Centre for Diabetes, Endocrinology and Metabolism (CEDAM), University of Birmingham, Birmingham, B15 2TT, United Kingdom; Centre for Membrane Proteins and Receptors (COMPARE), Universities of Birmingham and Nottingham, Birmingham, B15 2TT, United Kingdom

**Keywords:** calcium homeostasis, calcium-sensing receptor, g protein coupling, hypoparathyroidism, short stature

## Abstract

Autosomal dominant hypocalcemia (ADH) is due to enhanced calcium-dependent signaling caused by heterozygous gain-of-function (GOF) variants in the *CASR* gene (ADH1) or in the *GNA11* gene, encoding Gα11 (ADH2). Both ADH1 and ADH2 are associated with hypocalcemia and normal or inappropriately low levels of circulating PTH. ADH1 patients typically manifest hypercalciuria, while ADH2 is associated with short stature in approximately 42% of cases. We evaluated a 10-yr-old boy with hypoparathyroidism and short stature. Biochemical analyses revealed hypocalcemia, hyperphosphatemia, and inconsistent hypercalciuria. Genetic analyses revealed a de novo heterozygous p.Leu723Arg variant in *CASR.* We characterized the expression of recombinant WT and Leu723Arg calcium-sensing receptor (CaSR) proteins in HEK293 cells and assessed G protein activation in vitro by CaSR using bioluminescence resonance energy transfer. Transient expression studies showed the Leu723Arg variant was normally expressed but resulted in a significantly lower EC_50_ for extracellular calcium activation of G11 but not other G proteins (ie, Gi, Gq, Gs). The Leu723Arg substitution has a novel GOF phenotype that leads to biased CaSR activation of G11 signaling, suggesting that residue 723 specifies activation of G11 but not other G proteins. Similar studies of a previously described CaSR variant associated with hypoparathyroidism and short stature, Leu616Val, showed no changes in any G protein pathways, indicating it is likely to be a benign variant. Given the preferential activation of G11 by the Leu723Arg CaSR variant, we propose that the patient’s short stature shares a similar basis to that in patients with ADH2 due to GOF variants in *GNA11*.

## Introduction

G protein-coupled receptors (GPCRs) mediate numerous endocrine actions including growth, appetite, reproduction, bone and mineral metabolism, and glucose homeostasis by coupling to heterotrimeric guanine nucleotide-binding proteins (G proteins) that bind and hydrolyze GTP. G proteins consist of a Gβγ dimer lipid-anchored to plasma membranes and a distinctive α subunit that is a member of 4 major classes: Gαs (G-stimulatory), Gαi/o (G-inhibitory), Gαq/11, and Gα12/13. Ligand activation of GPCRs enables the receptor to induce conformational changes in heterotrimeric G proteins that facilitate GDP-to-GTP exchange. This exchange triggers dissociation of Gα from Gβγ (and the receptor) with both Gα-GTP and Gβγ then free to activate signaling cascades. The specificity of the signal response is primarily determined by the Gα class, which exhibits preferential interaction with various effector molecules. Because many GPCRs, including the calcium-sensing receptor (CaSR),^1^^,^^2^ couple to more than one G protein,^3^ G protein-coupled signal transduction can elicit highly complex responses. The CaSR is widely expressed but has a special role in mineral metabolism through its abundant expression on parathyroid cells and renal tubular epithelium, where it regulates PTH release and urinary calcium excretion, respectively. Interaction of extracellular calcium (Ca^2+^_e_) with the CaSR extracellular domain activates Gq/11 to stimulate phospholipase-C (PLCβ). PLCβ catalyzes phosphatidylinositol 4,5-bisphosphate (PIP2) hydrolysis to produce inositol 1,4,5-trisphosphate (IP3) and diacylglycerol (DAG). IP3 binds to receptors on the endoplasmic reticulum, thereby releasing stored intracellular calcium (Ca^2+^_i_) into the cytosol. DAG stimulates protein kinase-C, which activates MAPK pathways. CaSR also activates MAPK pathways through Gi/o-dependent inhibition of adenylyl cyclase.^4^ Some cell contexts facilitate CaSR activation of G12/13 and Gs^4^, altering cell growth and proliferation and stimulating adenylyl cyclase, respectively. The mechanisms that enable CaSR to manifest selectivity in G protein interaction are beginning to be elucidated, with cryo-EM structures suggesting that CaSR adopts substantially different structural conformations with distinct CaSR-G protein interfaces that enable interaction with different G proteins.^2^ These models indicate that selective coupling to Gq or Gi proteins relies on distinct interactions with residues in CaSR intracellular loop (ICL)-2 and -3 and the cytoplasmic C-tail, consistent with previous mutagenesis studies.^5^^,^^6^ This suggests that disruption of any residues in CaSR that mediate distinct binding with Gq or Gi are likely to result in biased signaling profiles.

Heterozygous germline mutations of the *CASR* that lead to a gain-of-function (GOF) cause autosomal dominant hypocalcemia type-1 (ADH1).^7^ Many ADH1 mutations cluster in regions involved in receptor activation, including transmembrane helix (TM)-6, while some ADH1-causing mutations bias signaling to couple more strongly to Ca^2+^_i_ mobilization than to ERK1/2 phosphorylation and other signaling pathways, and an Arg680Gly mutant activates a G protein-independent β-arrestin pathway.^8^^,^^9^ The identification of individuals harboring *GNA11* mutations with ADH2 supports the physiological activation of PLCβ signaling by CaSR.^10–12^ Two *GNA11* mutations that activate Gα11 and increase CaSR signaling are in the α5-helix,^10^^,^^13^ which facilitates GPCR activation of G proteins. Therefore, studying mutations in the CaSR intracellular region (ie, ICLs and TM residues proximal to ICLs) could provide insights into mechanisms by which CaSR activates different G proteins.

ADH1 and ADH2 are characterized by hypocalcemia, hyperphosphatemia, and normal or inappropriately low levels of circulating PTH.^7^ Increased fractional excretion of calcium by the kidney is typical of ADH1, while short stature is present in nearly half of patients with ADH2^12^^,^^14–16^ (Table S1). These phenotypic differences are thought to be due to variations in relative expression of the G proteins involved in CaSR signaling in different tissues (ie, G11 in the parathyroid and growth plate and Gq in the kidney). A spontaneous mouse model of ADH1, the *Gprc2a^Nuf^* mouse (referred to as *Nuf*), has a GOF Leu723Gln *Casr* mutation and manifests hypocalcemia, hyperphosphatemia, reduced serum PTH and ectopic calcifications most notably in the ocular lens, but lacks hypercalciuria.^17^ Subsequent knock-in mouse models of ADH1 mutations described mice with a smaller body size and low bone mineral density,^18^ which are not common features of human ADH1.^7^ In contrast, short stature has been reported in 5 cases or families with ADH2^12^^,^^15^^,^^16^^,^^19^^,^^20^ (approximately 42% of all reported mutations) (Table S1), while 2 mouse models with activating *Gna11* mutations have growth defects and/or changes in bone morphology (reduced bone mineral density).^21^^,^^22^ Studies indicate that G11 has an important role in growth^14^^,^^23^^,^^24^ likely via activation by PTH1R as well as CaSR in chondrocytes.^25^

We describe a boy with ADH1 and short stature who carries a novel *CASR* variant, Leu723Arg, in the homologous amino acid (Leu723) as the *Nuf* mouse, which has a Leu723Gln mutation. Our functional studies showed his unusual phenotype may be due to biased GOF induced by the Leu723Arg substitution that specifically enhances G11 activation, without increasing sensitivity to activation for other G proteins.

## Materials and methods

### Study approval

The patient and parents provided informed consent to genetic studies and enrolled in the global multi-center disease monitoring study of ADH1 and ADH2 (NCT05227287, Calcilytix Therapeutics, Inc). The Invitae hypoparathyroidism panel (PR06002.08) analyses 18 genes associated with isolated or syndromic hypoparathyroidism (*AIRE, CASR, CHD7, CYP24A1, FAM111A, GATA3, GCM2, GNA11, GNAS, HADHA, HADHB, PDE4D, PTH, PTH1R, SLC34A1, STX16, TBCE, TBX1*).

### Protein sequence alignment and 3-dimensional modeling of CaSR structure

Protein sequences of CaSR orthologs (human, rat, mouse, xenopus, zebrafish) were aligned using ClustalOmega.^26^ SIFT, Polyphen2, and MutationTaster were used to predict the effect of amino acid substitutions. MutationTaster (https://www.mutationtaster.org/index.html) predicts pathogenicity based primarily on evolutionary conservation.^27^ Polyphen-2 (http://genetics.bwh.harvard.edu/pph2/) predicts pathogenicity based on 8 sequence-based and 3 structure-based predictive features.^28^ Sorting Intolerant from Tolerant (SIFT) (https://sift.bii.a-star.edu.sg/) predicts whether an amino acid substitution affects protein function based on sequence homology and amino acid physical properties.^29^

CaSR 3-dimensional modeling was undertaken using the reported 3-dimensional cryo-EM structures (Protein Data Bank (PDB) accession numbers: 7E6T, 7R6U, 7SIM, 7SIL, 7SIN, 7M3F, 7M3G, 7M3J, 7M3E, 8SZF, 8SFG, 8SZH, 8SZI^2^^,^^30–32^) in the PyMOL Molecular Graphics System (Version 2.5.2, Schrodinger, LLC) (Table S1). Structures of CaSR in complex with Gαq were obtained with a chimeric construct encoding the N-terminus of Gαi1 (amino acids 1-28) and the C-terminus of Gαq.^2^ Therefore, contacts with Gαq-αN may be unreliable. Modeling of CaSR in complex with other G proteins was performed by AlphaFold2 using the ColabFold v1.5.2-patch in Google Co-laboratory^33^ with FASTA sequences obtained from NCBI. Five models were predicted and ranked based on predicted local distance difference test and the model with the highest confidence and correct orientation used for predictions. Modeling with Gα11 was performed first as it is highly expressed in the parathyroids and is mutated in ADH2.^1^^,^^10^^,^^11^ This yielded 4 structures with the correct orientation. Alphafold2 predictions were also performed for CaSR with Gαi1 (the most highly expressed Gαi/o protein in the parathyroid),^1^ and Gα12, Gα13, and Gαs. All 5 Gα12 and 2 Gα13 models were in the incorrect orientation, and these were therefore discarded. The models with the highest confidence measure were selected for assessment of the effect of the ADH1-associated Arg723 variant. All models showed Leu723 at the cytoplasmic end of TM4 and identified the interactions with the adjacent Gly720 and Leu727, which are retained by the Arg723 variant residue.

### Cell culture and transfection

Signaling studies were performed in Adherent HEK293 (AdHEK) cells as there are no good parathyroid cell-lines that are widely available; these cells do not endogenously express CaSR and they have been used by multiple research groups so findings can be compared easily. AdHEK cells (Agilent Technologies, Santa Clara, CA, United States) were maintained in DMEM-Glutamax media with 10% FBS (Sigma, Gillingham, United Kingdom) at 37 °C, 5% CO_2_. Cells were routinely screened to ensure they were mycoplasma-free. The ADH1-associated Arg723 and non-ADH1-associated Ala723 variants were introduced into a CMV-FLAG-CaSR plasmid and 3 previously reported ADH1 mutations (Gln27Glu, Pro221Leu, Thr828Asn)^34–36^ introduced into a CMV-CaSR plasmid by site-directed mutagenesis using the Quikchange Lightning Kit (Agilent Technologies) and oligonucleotides from Sigma. Mutations were confirmed by Sanger sequencing (Source Bioscience). Expression constructs were transiently transfected into cells using Lipofectamine 2000 (LifeTechnologies, Carlsbad, CA). Plasmids are detailed in Table S3. For studies with siRNA, cells were co-transfected with a combination of 3 different siRNA constructs targeting *GNA11* ((Trilencer-27 siRNA kit, catalog number SR301839, Origene) or *GNAQ* (Trilencer-27 universal scrambled negative control siRNA duplex, catalog number SR30004, Origene).

### Assessment of CaSR expression

For confocal microscopy, immunoblot analysis and ELISA, AdHEK cells were plated in 6-well plates and transfected with 1 μg of CaSR plasmid DNA per well 24-h later. For confocal microscopy, cells were fixed after a further 24-h, permeabilized, immunostained with primary anti-CaSR (mouse monoclonal, clone 5C10, ADD, 1:250 dilution, Abcam, Cambridge, United Kingdom) and secondary antibody Alexa Fluor 488 (1:500 dilution, Molecular Probes, Carlsbad, CA, United States), before mounting in Prolong Gold Antifade reagent (Invitrogen) and imaging by confocal microscopy using a Zeiss LSM780 with a Plan-Apochromat x63/1.2/water DIC objective and an argon laser.

Immunoblot analysis to assess expression of transfected CaSR was performed 48-h after transfection, with endogenous calnexin used as a loading control, using previously described methods.^1^ For ELISA, cells were replated in 96-well plates 48-h after transfection. Cells were left to settle for at least 4 h, then fixed in 4% paraformaldehyde/PBS (Sigma). Following blocking in serum, cells were incubated with an anti-CaSR antibody (1:500 dilution, ADD) for 3 h at room temperature, then an alkaline phosphatase conjugated secondary antibody for an hour. Plates were washed in PBS-t, then para-nitrophenyl phosphate (pNPP, ThermoFisher) added as a substrate for alkaline phosphatase, and absorbance read at 405 nm on a Glomax (Promega, Southampton, United Kingdom) plate reader.

### Bioluminescence resonance energy transfer G protein activation assay

To assess G protein signaling in real-time, we used a bioluminescence resonance energy transfer (BRET) assay that monitors GPCR-induced dissociation of Gα from βγ^3^. Upon Gα activation, Gβγ dimers fused to a Venus fluorescent protein (comprising 2 constructs, Venus-156-239-Gβ and Venus-1-155-Gγ, that form the Venus protein by bimolecular fluorescence complementation) dissociate from Gα and bind to G protein receptor kinase-3 tagged to Nanoluciferase (GRK3-Nluc), the BRET donor. G protein activation BRET assays were performed using methods adapted from previous studies.^3^^,^^37^ AdHEK cells were seeded at 100 000 cells/well in 6-well plates and transfected 24 h later with 50 ng of masGRK3ct-Nluc, Venus-156-239-Gβ1, Venus-1-55-Gγ2, 300 ng of pCMV-FLAG-CaSR-WT or CaSR variants and 100 ng untagged G proteins (either pIRES-puro-Gα11, pcDNA3.1-Gαq, -Gαi1, -Gαs). Forty-eight hours later, cells were washed with PBS, then detached and resuspended in FluoroBrite phenol red-free complete media (with 10% FBS and 2 mM L-Glutamine, ThermoFisher) and plated across 8-wells in a white 96-well microplate. At least 4 h later, media was replaced with calcium- and magnesium-free Hanks buffered saline solution (HBSS) and incubated for at least 30 min. Nano-Glo reagent (furimazine, Promega) was added at a 1:100 dilution and BRET measurements recorded using a PHERAstar (BMG Labtech, Aylesbury, United Kingdom) microplate reader (donor wavelength 475-30, acceptor wavelength 535-30) at 37 °C. Four baseline recordings (0, 2, 4, 6 min) were made, then agonist added at 8 min and recordings made for a further ~50 min. The BRET ratio (acceptor/donor) was calculated for each time point, the average baseline value subtracted from the experimental BRET signal, then responses normalized to vehicle to obtain the Normalized BRET ratio. AUC was calculated in GraphPad Prism and used to plot concentration-response curves with a 4-parameter sigmoidal fit.

### Glosensor cAMP assays

AdHEK cells were plated in 6-well plates and transfected with 100 ng pGloSensor-20F plasmid and either 500 ng pCMV-FLAG-CaSR-Leu723 (WT) or pCMV-FLAG-CaSR-Arg723. Forty-eight hours later, cells were seeded in 96-well plates in FluoroBrite complete media (ThermoScientific). Cells were incubated for at least 4 h, then media changed to 100 μL of equilibration media consisting of Ca^2+^- and Mg^2+^-free HBSS containing a 2% (v/v) dilution of the GloSensor cAMP Reagent stock solution. Cells were incubated for 2 h at 37 °C. Basal luminescence was read on a Glomax plate reader for 8 min, then agonist added with 10 μM forskolin (Sigma), and plates read for a further 30 min. Data were plotted in GraphPad Prism, area-within-the-curve calculated and these values used to plot concentration-response curves with a 4-parameter sigmoidal fit.

### IP3 biosensor assays

IP3 biosensor assays were performed as described.^38^ AdHEK cells were seeded at 10 000 cells/well in 96-well plates and transfected 8 h later with 200 ng LgBiT-IP3R2-SmBiT, either 500 ng pCMV-FLAG-CaSR-Leu723 (WT) or pCMV-FLAG-CaSR-Arg723 and scrambled, *GNA11*-targeting or *GNAQ-*targeting siRNA (Origene). Forty-eight hours later, media was changed to calcium- and magnesium-free HBSS and incubated for at least 1 h. Nano-Glo reagent (furimazine, Promega) was added at a 1:100 dilution and incubated for 10 min to allow stabilization of the luminescent signal. Luminescence was measured on a Glomax plate reader. Four baseline recordings (0, 2, 4, 6 min) were made, then agonist added at 8 min and recordings made for a further ~50 min. The average baseline value was subtracted from the experimental signal, then responses normalized to vehicle. AUC was calculated in GraphPad Prism and used to plot concentration-response curves with a 4-parameter sigmoidal fit.

## Results

### Identification of a *CASR-Leu723Arg* variant in a patient with ADH1 and short stature

The proband presented for evaluation of short stature to 1 author (MRB) at age 10 yr and 10 mo. He was born in Venezuela to non-consanguineous parents at full term with normal weight (*Z*-score, +0.3 WHO) and reduced length of 34 cm (*Z*-score, −8.39 WHO). Complete medical records were not available, but the mother reported that at age 3 mo while still in Venezuela, poor growth led to the diagnosis of failure to thrive and renal tubular acidosis (RTA) and treatment with bicarbonate supplements began. This led to some improvement in his growth and bicarbonate treatment was discontinued at age 3 yr. At age 10 yr and 10 mo he was evaluated in the United States by a nephrologist who found no evidence of RTA. The patient had proportionate short stature of 129.5 cm (*Z*-score, −1.98), with a weight of 26.5 kg (*Z*-score, −1.8). His bone age at 9 yr was delayed (*Z*-score 2.8) and his predicted height was 164.7 ± 5 cm compared with his mid-parental target height (MPTH) of 171.5 ± 10 cm. His most recent bone age at 14 yr 5 mo was normal (*Z*-score, −0.2) and his predicted adult height was 166.6 ± 5 cm. At age 14 yr and 5 mo he is now in late puberty growing along the 6.8 percentile for height (CDCZ-score, −1.49) with a height of 154.5 cm and a normal growth velocity of 9.5 cm/yr (Figure S1). Biochemical evaluation revealed mildly reduced serum calcium, elevated serum phosphorus, and reduced serum PTH (Table 1). A renal ultrasound was normal, and there was no history of nephrolithiasis. DNA analysis using a commercial next generation panel (Invitae hypoparathyroidism panel (PR06002.08)) revealed a heterozygous missense variant in the *CASR* gene (c.2168 T > G, p.Leu723Arg) that was not present in his parents.

**Table 1:** Serum calcium, phosphorous PTH, and urine chemistry over time.

Treatment	Age	Calcium mmol/L (mg/dL)	PTH (pg/mL)	Phosphorus mmol/L (mg/dL)	FECa^a^	24-h urine calcium (mg/24 h)	Urine volume (mL/24 h)
None	10 yr 11 mo	2.05 (8.2)	6	2.29 (7.1)	-	-	-
None	11 yr 1 mo	2.07 (8.3)	9	2.68 (8.3)	-	410	2000
None	11 yr 11 mo	1.93 (7.8)	13	2.22 (6.9)	-	92	1050
None	13 yr 5 mo	2.13 (8.5)	-	2.55 (7.9)	0.48 %	51.5	1700
None	13 yr 10 mo	2.02 (8.1)	-	2.65 (8.2)	0.62 %	98.8	1900
None	14 yr 5 mo	2.05 (8.2)	-	2.49 (7.7)	0.39 %	50.6	2300
Amiloride/HCTZ^b^	11 yr 2 mo	2.17 (8.7)	-	2.64 (8.2)	-	67	700
Amiloride/HCTZ	11 yr 6 mo	2.07 (8.3)	-	2.22 (6.9)	-	-	-
Amiloride/HCTZ	11 yr 11 mo	1.95 (7.8)	13	2.22 (6.9)	-	-	-
Amiloride/HCTZ	12 yr 4 mo	2.1 (8.4)	15	2.48 (7.7)	-	49^*^	400^c^
Calcitriol^d^	12 yr 10 mo	2.12 (8.5)	-	2.61 (8.1)	-	108	1000
Normal range for age		2.22-2.59 (8.9-10.4)	14-85	0.97-1.94 (3-6)		55-300 mg/24 h	≤500 mL/24 h deemed an inadequate urine collection volume for children <16 yr of age

Magnesium measured multiple times and always normal (1.5-2.5 mg/dL), with a reference range of 1.9-2.1 mg/dL. High urinary calcium was only observed upon first measurement. A diuretic treatment was tried after this although adherence was inconsistent and treatment was stopped. Urinary calcium excretion was not found to be elevated in subsequent analyses.

a FECa, fractional excretion of calcium.

b Given as Moduretic comprising 5 mg Amiloride and 50 mg Hydrochlorothiazide (HCTZ).

c Inadequate 24-h urine collection (Urine collection should be ≥500 mL/24 h for age).

d Calcitriol 0.5 mcg daily and calcium carbonate 1250 mg daily.

Because of initial hypercalciuria, the patient was treated with 2.5 mg of amiloride plus 25 mg of hydrochlorothiazide daily. After a month, the dose was doubled but adherence to therapy was inconsistent. Amiloride and hydrochlorothiazide were subsequently discontinued as hypercalciuria was no longer present off therapy and calcitriol (0.5 mcg daily) and 500 mg of elemental calcium carbonate daily were prescribed with little changes in his serum calcium and urinary calcium excretion (Table 1). Therapy was discontinued after 5 mo due to poor adherence and lack of symptoms. The fractional excretion of calcium has been low (0.01%) and renal ultrasound did not reveal any calcifications. He had cerebral calcifications on CT scan of the head with noted dense, symmetric calcifications demonstrated in the bilateral lentiform nuclei and bilateral frontal juxta-cortical white matter. Faint calcifications were also present in the bilateral caudate nuclei.

### Characterization of the *CASR-Leu723Arg* variant

**Figure 1:**
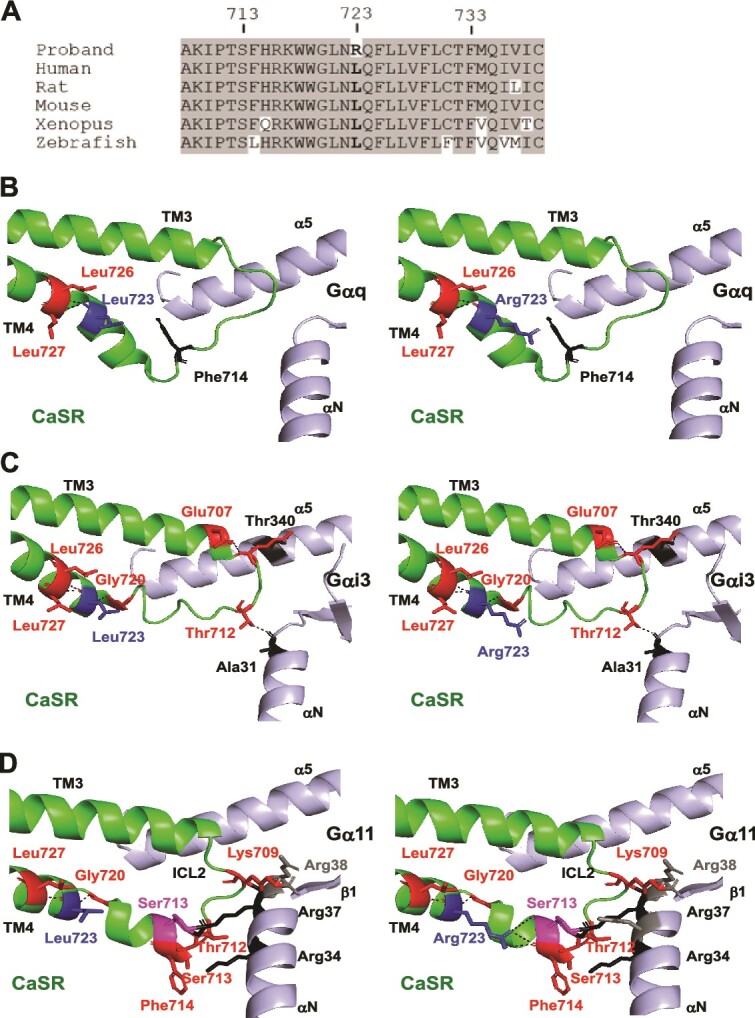
Identification of an ADH1-associated Arg723 CaSR variant within the G protein binding region. (A) Multiple protein sequence alignment of CaSR orthologs in 5 species. Conserved residues are shown in gray. Residue 723 is in bold. (B) Three-dimensional model of the CaSR-Gαq interface (PDB ID: 8SZG)^2^ and (C) the CaSR-Gαi3 interface comprising CaSR ICL2, and αN and α5 of the G protein (PDB ID: 8SZI).^2^ Residue 723 is shown in blue, with (left) the WT Leu723 and (right) variant Arg723. Residue 723 forms contacts (black hatched lines) with Leu726 and Leu727, which are retained by Arg723. The variant Arg723 side chain projects closer to ICL2 and could affect the CaSR ICL2-Gαq interface but is predicted to have no effect on Gαi3 binding. (D) AlphaFold2 model of the CaSR-Gα11 interface showing CaSR ICL2 and Gα11-αN and α5. The Arg723 variant forms additional contacts with Ser713 and Phe714, which may affect CaSR-Gα11 activation.

The de novo T-to-G transversion at nucleotide c.2168 (c.2168 T > G) resulted in a missense substitution, p.Leu723Arg, in the CaSR protein. Multiple lines of evidence indicate that replacement of Leu723 by Arg is pathogenic. First, bioinformatic analyses using MutationTaster2 and Polyphen-2 software predicted the p.Leu723Arg variant to be disease-causing and probably damaging, respectively, although SIFT predicted the variant may be tolerated. Second, ClustalOmega analysis revealed the Leu723 WT residue is highly evolutionarily conserved across CaSR orthologs (Figure 1A). Third, the p.Leu723Arg variant was not present in the Genome Aggregation Database (GnomAD). And fourth, a similar Leu723Gln missense mutation has previously been reported as pathogenic in the *Nuf* mouse, a model for ADH1.^17^ Therefore, it is likely that replacement of Leu723, either to Arg723 as observed in the ADH1 patient or to Gln723 as observed in the *Nuf* mouse, has a functional effect on CaSR.

### Structural characterization of the CaSR-Leu723Arg variant protein

The location of the Leu723 WT residue, and predicted effects of the ADH1-associated Arg723 variant, were investigated in published cryo-EM models^2^^,^^30–32^ with PDB codes listed in Table S2. In all models, Leu723 contacts adjacent non-polar residues, but no contacts were disrupted by the ADH1-associated Arg723 variant (Table S2). Models of CaSR in complex with Gq and Gi3 show Leu723 adjacent to the ICL2-G protein binding region. Arg723 is not predicted to affect Gq or Gi3 binding. However, the Arg723 variant side chain projects toward ICL2 in the CaSR-Gq model, which could affect the GPCR-G protein binding interface (Figure 1B and C). AlphaFold2 was used to generate models with other Gα proteins to determine whether Arg723 may affect their coupling. All models predicted Leu723 within TM4 adjacent to the G protein binding site. The CaSR-Gα11 model predicted the variant Arg723 forms several contacts with Phe714 within the CaSR ICL2-Gα11 interface, which are absent in the WT and could affect CaSR-Gα11 activation (Figure 1D). In the Gαi1 model, a single new contact is predicted between Arg723 and Phe714 (Figure S2), indicating that the Arg723 variant may have a similar effect on Gαi1 coupling. No new contacts are formed in Gα13 and Gαs models (Figure S2). Therefore, modeling predicts that the Arg723 variant is likely to affect Gα11 signaling and may affect Gαi1, but not activation of Gαq or other G proteins.

### The ADH1-associated Arg723 CaSR variant enhances Gα11 signaling

**Figure 2:**
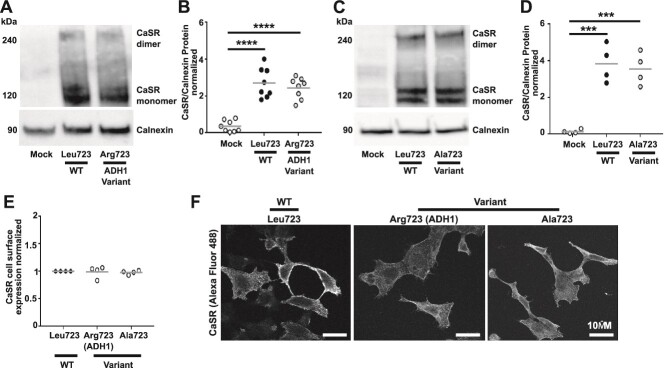
Mutation of the Leu723 to Arg723 has no effect on CaSR protein or cell surface expression. (A) Immunoblot analyses of CaSR in HEK293 cells transfected with WT (Leu723) and the ADH1-associated variant Arg723 compared with mock transfected cells. Endogenous calnexin was used as a loading control. (B) Densitometry analysis of CaSR expression levels in immunoblots from 8 biological replicates. Data were normalized to endogenous calnexin. (C) Immunoblot analyses of CaSR in HEK293 cells transfected with WT (Leu723) and the non-ADH1-associated variant Ala723 compared with mock transfected cells. (D) Densitometry analysis of CaSR expression levels in immunoblots from 4 biological replicates. (E) CaSR cell surface expression measured by ELISA in 4 biological replicates. Data were normalized to the expression of WT CaSR. (F) Immunofluorescence analyses showing cell surface and cytoplasmic expression of CaSR in WT and Arg723 or Ala723 variant expressing cells. Statistical analyses were performed by 1-way ANOVA with Tukey test for panels B and D, and 1-way ANOVA with Holm-Sidak test for E.

**Figure 3:**
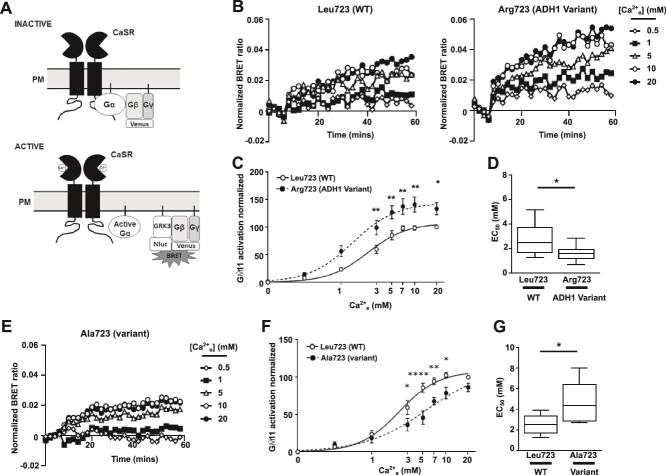
The ADH1-associated Arg723 CaSR variant enhances Gα11 responses. (A) Cartoon demonstrating the G protein activation assay used in these studies. Gγ and Gβ are tagged with the N- and C-terminal parts of the fluorescent Venus protein, respectively, and Gβγ dimer formation allows bimolecular fluorescence complementation. Upon agonist-induced stimulation of the CaSR, Gβγ dissociates from the activated Gα protein and associates with GRK3 tagged to Nluc, which serves as a BRET donor. (B) Representative traces of BRET ratios measured between GRK3-Nluc and Venus-Gβγ in AdHEK cells transfected with Gα11 and CaSR WT Leu723 (WT) and the ADH1-associated Arg723 variant showing 5 [Ca^2+^]_e_. (C) Dose-response generated from the AUC of the BRET responses with (D) EC_50_ with minimum and maximum values shown as error bars. *N* = 8 biological replicates. (E) Representative traces of BRET ratios between GRK3-Nluc and Venus-Gβγ in AdHEK cells transfected with the CaSR variant Ala723 showing 5 [Ca^2+^]_e_. (F) Dose-response generated from the AUC of the BRET responses with (G) EC_50_ with minimum and maximum values shown as error bars. *N* = 6 biological replicates. Statistical analyses were performed by 2-way ANOVA with Sidak test in C and F, and by unpaired *t*-test in D and G. ^****^p < .0001, ^**^p < .01, ^*^p < .05.

Immunoblot analyses, ELISA, and fluorescence microscopy confirmed similar total and cell surface expression of WT Leu723, ADH1-associated variant Arg723 CaSR or the non-ADH1-associated mutant Ala723 in transiently transfected AdHEK cells (Figure 2A-F). We assessed G protein signaling in real-time using a BRET assay that monitors GPCR-induced dissociation of Gα from Gβγ.^3^ Upon Gα activation, Gβγ dimers fused to a Venus fluorescent protein dissociate from Gα and bind to G protein receptor kinase-3 tagged to Nanoluciferase (GRK3-Nluc), the BRET donor (Figure 3A). AdHEK cells transfected with Venus-Gβγ, GRK3-Nluc, untagged Gα11 protein and either the CaSR WT Leu723, ADH1-associated variant Arg723 or non-ADH1-associated variant Ala723 proteins showed BRET responses increasing in a dose-dependent manner after stimulation with increasing Ca^2+^_e_ concentrations between 0-20 mM (Figure 3B). The area-under-the-curve of each BRET response was used to generate a concentration-response curve (Figure 3C), which demonstrated significantly reduced EC_50_ responses in cells expressing the ADH1-associated Arg723 variant when compared with WT Leu723 (Figure 3D), consistent with a GOF. The Ala723 variant reduced responses (Figure 3E-G).

### Biased signaling of the ADH1-associated Arg723 CaSR variant

**Figure 4:**
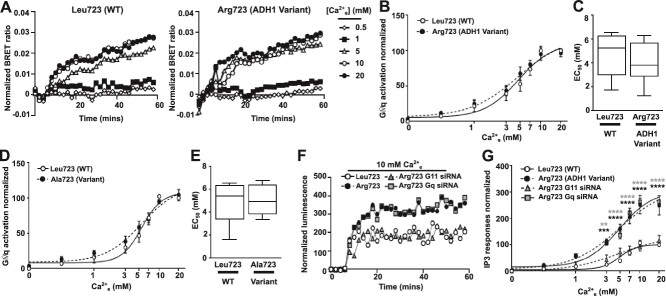
The ADH1-associated Arg723 variant has no effect on Gαq signaling. (A) Representative traces of BRET ratios measured between GRK3-Nluc and Venus-Gβγ in AdHEK cells transfected with Gαq and CaSR WT Leu723 (WT) and the ADH1-associated Arg723 variant showing 5 [Ca^2+^]_e_. (B) Dose-response generated from the AUC of the BRET responses with (C) EC_50_ with minimum and maximum values shown as error bars. *N* = 6 biological replicates. (D) Dose-response generated from the AUC of the BRET responses of cells transfected with Gαq and CaSR WT or the engineered mutant Ala723 with (E) EC_50_ with minimum and maximum values shown as error bars. *N* = 6 biological replicates. (F) Representative traces of luminescence normalized to baseline values in AdHEK cells transfected with an IP3 biosensor and CaSR WT or the Arg723 variant. Cells were co-transfected with scrambled, *GNAQ* or *GNA11* siRNA. (G) Dose-response generated from the AUC of the IP3 luminescence in cells transfected with siRNA normalized to WT. *N* = 5 biological replicates. Statistical analyses were performed by 2-way ANOVA in B, D, and G, and unpaired t-test in C and E. ^****^p < .0001. Data show comparisons with WT for Arg723 (with scrambled siRNA) in black and Arg723 (with *GNAQ* siRNA) in gray in panel G.

Our predicted structural models suggested that other G proteins may not interact with CaSR at the same site as Gα11, and therefore the ADH1-associated Arg723 variant may not affect coupling to these G proteins. We therefore used the same BRET G protein activation assay to assess interactions between CaSR and different G proteins. AdHEK cells transfected with GRK3-Nluc, Venus-Gβγ, untagged-Gαq and either Leu723 WT, the ADH1-associated Arg723 variant (Figure 4A-C), or Ala723 (Figure 4D and E) CaSR showed similar responses to all Ca^2+^_e_ concentrations and there was no change in maximal responses or EC_50_. These findings were confirmed by NanoBiT IP3 biosensor assays^1^ that showed the ADH1-associated Arg723 variant enhances responses (Figure 4F). Treatment with siRNA targeting *GNA11* reduced Arg723 responses such that they were not significantly different to WT expressing cells, while *GNAQ*-targeting siRNA did not reduce Arg723 responses (Figure 4F and G).

**Figure 5:**
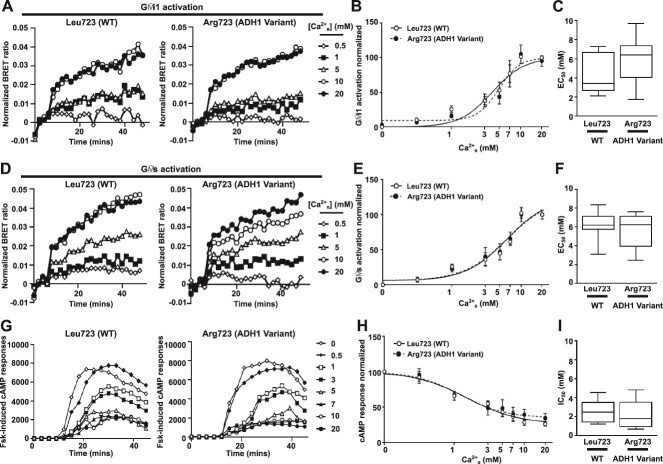
The ADH1-associated Arg723 variant has no effect on Gαi and Gαs signaling. (A) Representative traces of BRET ratios measured between GRK3-Nluc and Venus-Gβγ in AdHEK cells transfected with Gαi1 and CaSR WT Leu723 (WT) and the ADH1-associated Arg723 variant showing 5 [Ca^2+^]_e_. (B) Dose-response generated from the AUC of the BRET responses with (C) EC_50_ with minimum and maximum values shown as error bars. *N* = 7 biological replicates. (D) Representative traces of BRET ratios between GRK3-Nluc and Venus-Gβγ in AdHEK cells transfected with Gαs and CaSR WT Leu723 (WT) and the ADH1-associated Arg723 variant. (E) Dose-response generated from the AUC of the BRET responses with (F) EC_50_ with minimum and maximum values shown as error bars. *N* = 7 biological replicates. (G) Ca^2+^_e_-induced reductions in forskolin (Fsk)-generated cAMP measured in WT and Arg723 variant transfected cells. (H) Dose-response generated from the AUC of the cAMP Glosensor responses with (I) IC_50_ with minimum and maximum values shown as error bars. *N* = 5 biological replicates. Statistical analyses were performed by 2-way ANOVA with Sidak test in B, E, and H and unpaired *t*-test in C, F, and I.

We next assessed Gαi and Gαs responses as CaSR also couples to these pathways.^1^^,^^2^^,^^4^ In cells transfected with GRK3-Nluc, Venus-Gβγ and untagged-Gαi or untagged-Gαs there were no significant differences between Leu723 WT or ADH1-associated Arg723 variant (Figure 5A-F). To verify these findings, cAMP responses were also assessed using cAMP Glosensor and Ca^2+^_e_-mediated reductions in forskolin-induced cAMP. This confirmed cAMP responses in WT and Arg723 variant CaSR expressing cells were not significantly different (Figure 5G-I). An increase in cAMP responses was detected for the ADH1 mutant Leu173Phe, which is known to enhance both Gq/11 and Gi responses^9^ and served as a positive control (Figure S3). The CaSR variant, Ala723, also activated Gαi1 and Gαs but responses were not significantly different to WT CaSR (Figure S3). Finally, we examined 3 other ADH1 mutants (Gln27Glu, Pro221Leu and Thr828Asn) that have previously been shown to enhance Ca^2+^_i_ and/or ERK1/2 signaling.^34–36^ All 3 ADH1 mutants enhanced Ca^2+^-dependent activation of G11, Gq, Gi and Gs (Figure S4). Thus, substitution of the Leu723 residue affects CaSR coupling to G11, and not to Gi or Gs.

### The Leu616Val CaSR variant does not affect G protein activation

**Figure 6:**
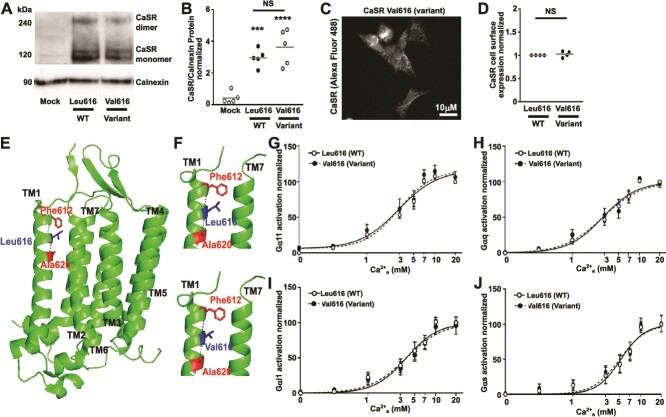
The CaSR Val616 variant does not affect G protein signaling. (A) Immunoblot analyses of CaSR in AdHEK cells transfected with WT (Leu616) or the ADH1-associated Val616 variant compared with mock transfected cells. Endogenous calnexin was used as a loading control. (B) Densitometry analysis of CaSR expression levels in immunoblots from 5 biological replicates. Data normalized to calnexin. (C) Immunofluorescence analyses showing cell surface and cytoplasmic expression of CaSR in the Val616 variant expressing cells. (D) CaSR cell surface expression measured by ELISA in 4 biological replicates. Data normalized to expression of WT CaSR. Statistical analyses by 1-way ANOVA with Holm-Sidak test. (E) Three-dimensional model of the CaSR transmembrane domain showing the location of the Leu616 residue in TM1 in blue (PDB ID: 8SZG). Contacts were the same in all structures from 4 published cryo-EM structures examined.^2^^,^^30–32^ (F) Close-up images show Leu616 forms contacts with the adjacent Phe612 and Ala620 residues. The Val616 variant, identified in a family with ADH1, retains these contacts. (G-J) Dose-response generated from the AUC of the BRET responses in AdHEK transfected with (G) Gα11, (H) Gαq, (I) Gαi1, (J) Gαs, and CaSR WT or the Val616 variant. *N* = 8 biological replicates for G and H, *N* = 7 for I and J. Statistical analyses performed by 2-way ANOVA with Sidak test in G-J.

The patient we identified with the CaSR Arg723 variant had hypoparathyroidism and short stature, but growth defects are not common in individuals with activating CaSR mutations. We performed a literature search for other cases that have been described as ADH1 with possible growth defects and identified 1 study describing a family with autosomal dominant hypoparathyroidism, short stature, and premature osteoarthritis,^39^ in which a CaSR variant, Leu616Val, had been identified to co-segregate with affected family members, but limited functional studies had not demonstrated a GOF. We sought to determine whether this Val616 variant also had a biased effect on G protein signaling. Total and cell surface expression were similar in cells transfected with Leu616 WT and Val616 variant CaSR (Figure 6A-D). Structural analyses showed Leu616 is in TM1 and forms contacts with adjacent residues that are not affected by the variant Val616 (Figure 6E and F). Transient transfection of either Leu616 WT or Val616 variant CaSR showed similar dose-dependent responses for Gα11, Gαq, Gαi1 and Gαs. Because there were no significant differences between WT or the Val616 variant (Figure 6G-J), we conclude that p.Leu616Val is likely a benign variant that has no effect on CaSR signaling. Therefore, the hypoparathyroidism and short stature described in this case are unlikely to be related to the CaSR Leu616Val variant and may be due to mutations in other genes.

## Discussion

We describe a child with ADH1 who carries a pathogenic *CASR* variant, Leu723Arg, that exhibits biased GOF activation of G11 and normal activation of other G proteins. Our functional analyses are consistent with a CaSR activating mutation, with a leftward shift in the Ca^2+^ dose-response for G11 and IP3 activation, reduced EC_50_, and elevated maximal responses. By contrast, alanine substitution of Leu723 has an opposite effect, impairing CaSR-mediated G11 activation. The *Nuf* mouse model of ADH1 has a mutation (Leu723Gln) in the homologous CaSR Leu723 residue that similarly results in enhanced CaSR signaling^17^ with biochemical features of hypoparathyroidism, including hypocalcemia, inappropriately low plasma PTH, and hyperphosphatemia.^17^ Therefore, the CaSR-Leu723 residue likely has a critical role within the ICL2 environment and contributes to Gα11 activation. Negative allosteric modulators (NAM) of the CaSR, NPS-2143 and NPSP795, rectify CaSR signaling in *Nuf* mice resulting in normalization of serum calcium and PTH.^40^^,^^41^ Studies showed that NPS-2143 can bias CaSR signaling, such that it preferentially favors coupling to Ca^2+^_i_ pathways.^8^ This effect has been attributed to changes in signaling efficacy, rather than changes in Ca^2+^_e_ affinity, and suggests NAM-bound CaSR adopts a structural conformation that impairs binding of some G proteins. Thus, calcilytic binding may allow CaSR to adopt a conformation in which the mutant Gln723 residue no longer sterically restricts ICL2, normalizing signaling to WT levels.

Our studies provide further evidence that CaSR may use distinct binding modes for different G proteins, consistent with cryo-EM models and, in line with mutagenesis studies, demonstrating the importance of CaSR-ICL2 in mediating coupling to different signaling pathways.^2^^,^^5^^,^^6^ A similar role for ICL2 in differential G protein signaling has been shown for another class C GPCR, mGluR1, indicating this may be a conserved mechanism.^42^ The ADH1-associated Arg723 variant is predicted to disrupt an interface between CaSR-ICL2 and the αN-β1 hinge and α5-helix regions of Gα11, which may favor a CaSR conformation that more readily induces coupling between CaSR and Gα11, or preferentially activates Gα11 compared with other G proteins. Mutations in the Gα11 αN-β1 hinge and α5-helix have been identified in ADH2 patients and shown to enhance CaSR activity^10^^,^^13^^,^^15^ and engineered mutations within this region reduce G protein affinity for GDP, favoring activation.^43^ Moreover, engineered mutations in the Gα binding residues of other GPCRs affect their G protein coupling.^3^ Therefore, other CaSR mutations in regions critical for differential G protein binding may also bias signaling.

Biased activation of G11, with normal stimulation of other G proteins, has not been reported for other CaSR ADH1 mutations and therefore appears to be a unique feature of the Arg723 variant. Specifically, we examined 3 previously reported^34–36^ ADH1 mutations, Gln27Glu, Pro221Leu and Thr828Asn, and found similarly enhanced activation of all G proteins examined. Therefore, biased signaling by G11 is not a common feature of all ADH1 mutations. However, most studies of CaSR variants do not examine G protein activation and instead measure downstream signaling (eg, ERK1/2 phosphorylation), which may be activated by multiple G protein pathways. Several ADH1-associated CaSR mutations have been described to couple more strongly to Ca^2+^_i_ mobilization than to other pathways,^8^ and it is possible these mutations may differentially activate distinct G proteins. Examining activation of individual G proteins by CaSR variants may reveal other CaSR residues with a similar G11-biased profile.

The individual we report here with the Arg723 CaSR variant presented with short stature, which is not a common feature of ADH1. A single case had been previously reported in which a variant in CaSR, Leu616Val, was associated with autosomal dominant hypoparathyroidism and short stature.^39^ In this previous study the authors described in vitro analyses to assess inositol phosphate accumulation in response to Ca^2+^ stimulation and found no differences between the Leu616Val variant and WT CaSR. Consistent with these previous studies, we were unable to identify differences in G protein activation between cells expressing WT or Val616 CaSRs and could not replicate the reduced CaSR expression noted previously (Figure 6). Therefore, it is likely this Leu616Val variant is a benign polymorphism incidentally identified in an individual with hypoparathyroidism. Reduced growth is not a feature of the *Nuf* mouse that has a Gln723 mutation in the Leu723 residue^17^; however, bone defects have been reported in a knock-in mouse of the Ala843Glu CaSR mutation,^18^ a recurrent mutation observed in several unrelated individuals with ADH1. The Glu843 knock-in mice had a smaller body and bone size, lower bone mineral density, and frequent microcracks, attributed to a negative calcium balance induced by the marked hypercalciuria of the mice.^18^ Hypercalciuria was noted once in our ADH1 patient but subsequent 24-h urine excretion and fractional excretion of calcium was normal, similar to that in ADH2.^11–13^^,^^15^

It is possible that the short stature observed in this individual with ADH1 may also be related to the biased activation of Gα11, compared with other G protein signaling pathways. The mechanism linking activating *GNA11* mutations to short stature is not yet determined but is likely to involve signaling through pathways linked to PTH1R and CaSR in the chondrocyte.^25^ PTHrP (or PTH) interaction with PTH1R expressed by epiphyseal chondrocytes activates multiple G proteins, including Gα11.^14^ The relative contributions of various G proteins to transducing PTH1R-dependent signals in chondrocytes are poorly understood, but Gαs appears to play the dominant role.^44^ Accordingly, studies have shown that PTH1R signaling through Gαs-cAMP prevents premature chondrocyte differentiation. Ablation of either Gαs or PTH1R leads to accelerated chondrocyte differentiation, and thus premature fusion of the growth plate postnatally and short stature. By contrast, the potential contribution of Gαq and Gα11 to chondrocyte differentiation remains poorly understood. One recent report has shown that a loss-of-function mutation in *Casr* could rescue the bone phenotype observed in *Pth* null mice by increasing osteoclast numbers and improving the columnar pattern of chondrocytes in the growth zone,^25^ thereby providing evidence of a direct effect of CaSR on chondrocytes. In addition, transgenic mice with Gα11 overexpression in osteoblasts showed decreased osteoanabolic effects of PTH and exercise compared with WT,^23^ while mice with the constitutively active Gα11-Gln209Leu mutation in osteoblasts had severe osteopenia in cortical and trabecular bones.^24^ Biased signaling through G11 would result in an imbalance of CaSR signaling such that a relative deficiency of Gi action compared with other ADH1 mutants could contribute to the short stature. Gi signaling at chondrocytes is not well understood.^44^ However, as Gi signaling by the Leu723Arg variant was not different to WT CaSR, we would assume that any physiological consequences of CaSR-Gi signaling would be similar for cells expressing either the Leu723Arg variant or WT receptor.

Our patient had short stature and initially a delayed bone age, features consistent with constitutional delay of growth and maturation. Later examinations revealed his bone age to be normal, however. Bone age has also been reported to be normal in ADH2 children with *GNA11* variants who had short stature and early intracerebral calcifications, similar to our patient.^15^ Additional reports of short stature in patients with ADH2 lend further support to the hypothesis that impaired long bone growth may be a consequence of increased signaling through Gα11-dependent pathways.^12^^,^^15^^,^^16^^,^^19^^,^^20^ although the effects of *GNA11* variants with increased activity are not entirely consistent (Table S1). The Val340Met Gα11 variant, located in the predicted ICL2-Gα11 binding site, is associated with short stature in 1 family,^15^ but normal height in another^13^; and similarly mutation of the adjacent residue Phe341Leu is associated with short stature in 1 family,^19^ but normal height in another.^10^ Additionally, mutation of Gα11-Arg60 to Leu60 is associated with short stature in 1 family,^14^ but members of 1 family with the Cys60 mutation have normal height,^11^ and a single case of Leu60Cys has short stature.^20^ Furthermore, the *Dsk7* mouse, which harbors a germline hypermorphic Gα11 mutation, and a knock-in mouse model with the Gα11-Arg60Cys mutation, have reduced body length and significantly lower BMD, respectively.^21^^,^^22^  *GNA11* mutations are associated with an overall mild increase in CaSR-mediated signaling compared with CaSR mutations^45^ and it is possible that this may influence the manifestation of symptoms in some cases of ADH. It is also not clear whether CaSR couples to multiple G proteins at chondrocytes which could also influence whether linear growth is affected in individual cases. Linear growth is a highly complex polygenic trait with more than 100 loci shown to influence human height.^46^ Therefore, some activating CaSR or Gα11 variants may contribute to genetic risk of short stature, but variants in other genes may influence whether height is affected in individual cases. The increased availability of whole-exome or whole-genome sequencing in diverse populations may help decipher the contribution that CaSR and Gα11 variants have on growth and height.

Transient hypercalciuria was observed in the patient harboring the Arg723 variant but fractional excretion of calcium was normal. On all other occasions the urinary calcium in the patient was not overtly increased but likely in the appropriate range given the low serum calcium. Hypercalciuria occurs in approximately one-third of ADH1 cases and typically during treatment when serum calcium levels are normal.^47^ By contrast, hypercalciuria is rare in ADH2, suggesting that renal CaSR may preferentially couple to Gq. Although renal expression of Gq and G11 appears similar,^1^ there may be important differences in cell type- or polarity-specific protein expression. Recent studies suggested that renal CaSR is less sensitive to activating mutations than parathyroid CaSR.^47^^,^^48^ Further studies of CaSR signaling in tissue-specific cells and greater numbers of patients with *GNA11* activating mutations may help answer these questions.

In conclusion, our studies demonstrated that the Leu723 residue of CaSR has a critical role in activating signaling via Gα11 but does not affect signaling through other Gα proteins. Activating mutations of this residue causes ADH1 in both humans and animals, providing further evidence of the importance of G11 signaling in CaSR function. Our studies have yielded insights into CaSR-G protein coupling selectivity, which may aid the design of novel therapeutic agents for targeting CaSR in hypo/hypercalcemic disorders.

## Supplementary Material

Revised_Supplementary_Appendix_Clean_zjae199

## Data Availability

All data needed to evaluate the conclusions in the paper are present in the paper and/or the Supplementary Materials. The number of experimental replicates denoted by n is indicated in figure legends. Data were plotted and statistical analyses performed in Graphpad Prism. Normality tests (Shapiro-Wilk or D’Agostino-Pearson) were performed on all datasets to determine whether parametric or non-parametric statistical tests were appropriate. A *p*-value of <.05 was considered statistically significant. Statistical analyses were performed as described in figure legends.
