# Supplementary material for: An activating calcium-sensing receptor variant with biased signaling reveals a critical residue for Gα11 coupling
Source: J Bone Miner Res. 2024 Dec 10;40(2):270–82. doi: 10.1093/jbmr/zjae199 (PMC11789390; doi:10.1093/jbmr/zjae199)
Supplement: Revised_Supplementary_Appendix_Clean_zjae199 [file revised_supplementary_appendix_clean_zjae199.docx]

**Supplementary Appendix**

**Table S1 Summary of all reported ADH2 cases showing the frequency of short stature**

| **Mutation** | **Short stature** | **Reference** |
| --- | --- | --- |
| Arg60Cys | No  Yes | Mannstadt *et al*^1^  Akad *et al*^2^ |
| Arg60Leu | Yes | Li *et al*^3^ |
| Gly66Ser | No | Gorvin *et al*^4^ |
| Arg149His | No | Gorvin *et al*^4^ |
| Arg181Gln | No | Nesbit *et al*^5^ |
| Arg183Pro | Yes | Kwon *et al*^6^ |
| Ser211Trp | No | Mannstadt *et al*^1^ |
| Val340Trp | No  Yes | Piret *et al*^7^  Tenhola *et al*^8^ |
| Phe341Leu | No  Yes | Nesbit *et al*^5^  Takahashi *et al*^9^ |

Short stature has been reported in 5 of 12 cases and therefore has a frequency of approximately 42% in ADH2 cases.

**Table S2 Assessment of the Leu723 wild-type and Arg723 variant residue in CaSR cryo-EM structures**

| **Reference** | **Protein Data Bank code** | **State** | **Location of Leu723** | **Leu723** | **Arg727** |
| --- | --- | --- | --- | --- | --- |
| **Chen *et al*^10^** | 7E6T | Active (Ca^2+^-TNCA) | ICL2 | Contacts with Leu726 (TM4) & Leu727 (TM4). | Contacts retained |
|  | 7E6U | Inactive, Negative allosteric nanobody (NB2D11) | TM4 | Contacts with Gly720 (TM4) & Leu727 (TM4). | Contacts retained, new contact formed with Phe706 |
| **Park *et al*^11^** | 7SIM | Active (Ca^2+^-TNCA) | Last residue of TM4 | Contact with Leu727 (TM4). | Contact retained |
|  | 7SIL | Active (Ca^2+^-TNCA), PAM (R-568) | Last residue of TM4 | Contact with Leu727 (TM4). | Contact retained |
|  | 7SIN | Inactive (inhibitory anion sulfate, ethylene glycol, EGTA), NAM (NPS-2143) | Last residue of TM4 | Contact with Leu727 (TM4). | Contact retained |
| **Gao *et al*^12^** | 7M3F | Active (Ca^2+^-Trp), PAM (cinacalcet) | ICL2 | No contacts. | No contacts |
|  | 7M3G | Active (Ca^2+^-Trp), PAM (etelcalcetide–evocalcet) | ICL2 | Contact with Leu727 (TM4). | Contacts retained |
|  | 7M3J | Inactive, NAM (NPS-2143) | TM4 | Protomer 1: contacts with Leu726 (TM4) & Leu727 (TM4).  Protomer 2: contacts with Leu721 (ICL2) & Leu727 (TM4). | Contacts retained |
|  | 7M3E | Active (Ca^2+^–Trp), NAM (NPS2143) | TM4 | Contact with Leu727 (TM4), contact with Leu704 (ICL2). | Contacts retained |
| **He *et al*^13^** | 8SZF | Active (Ca^2+^, Trp), PAM (cinacalcet) | TM4 | Contact with Leu726 (TM4) and Leu727 (TM4). | Contacts retained |
|  | 8SZG | PAM (cinacalcet) bound, in complex with Gq | TM4 | Contact with Leu726 and Leu727. | Contacts retained.  Closer proximity to α5-helix (4Å distance) |
|  | 8SZH | Active (Ca^2+^, Trp), PAM (cinacalcet), in complex with Gi | TM4 | Contact with Gly720 (ICL2) and Leu727 (TM4). | Contacts retained, new contact formed with Lys717 (ICL2). |
|  | 8SZI | Active (Ca^2+^, Trp), in complex with Gi | TM4 | Contact with Gly720 (ICL2), Leu726 (TM4) and Leu727 (TM4). | Contacts retained. |

Structures from Ling *et al ^14^* did not contain residue Leu723, therefore these structures were not included in the table. TNCA is a tryptophan analog, L-1,2,3,4-tetrahydronorharman-3-carboxylic acid.

# Table S3 Expression plasmids used in this manuscript

| **Plasmid name** |  | **Information** |  | **Source** |
| --- | --- | --- | --- | --- |
| pCMV-FLAG-CaSR |  | Full-length *CASR* with an N-terminal FLAG tag and CMV promoter |  | Custom order from Twist Bioscience, San Francisco, CA |
| pCMV-FLAG-CaSR-Leu723Arg |  | Full-length *CASR* with an N-terminal FLAG tag with the Leu723Arg variant |  | This manuscript |
| pcDNA3.1-FLAG-CaSR-WT |  | Full-length *CASR* with an N-terminal FLAG tag |  | Caroline Gorvin, University of Birmingham^14^ |
| pcDNA3.1-FLAG-CaSR-Leu723Ala |  | Full-length *CASR* with an N-terminal FLAG tag with the Leu723Ala variant |  | This manuscript |
| pcDNA3.1-FLAG-CaSR-Leu173Phe |  | Full-length *CASR* with an N-terminal FLAG tag with the Leu173Phe variant |  | This manuscript |
| pIRES-puro-GNA11 |  | WT *GNA11* (Gα11) sequence |  | This manuscript |
| pcDNA3.1-GNAQ |  | WT *GNAQ* (Gαq) sequence |  | Joachim Goedhart (University of Amsterdam, The Netherlands) |
| pcDNA3.1-GNAI1 |  | WT *GNAI1* (GαI1) sequence |  | This manuscript |
| pcDNA3.1-GNAS |  | WT *GNAQ* (Gαs) sequence |  | This manuscript |
| Venus-1-55-Gγ2 |  | BRET; bimolecular fluorescence complementation (BiFC)-based BRET acceptor |  | Nevin Lambert, Augusta University*^15,16^* |
| Venus-156-239-Gβ1 |  | BRET; bimolecular fluorescence complementation (BiFC)-based BRET acceptor |  | Nevin Lambert, Augusta University*^15,16^* |
| masGRK3ct-Nluc |  | BRET; membrane-associated C-terminal fragment of the GRK3 |  | Nevin Lambert, Augusta University*^15,16^* |
| cAMP Glosensor-22F |  | cAMP sensor |  | Promega |
| LgBiT-IP3R2-SmBiT |  | IP_3_ biosensor |  | Asuka Inoue, Tohoku University^17^ |
| pCMV-FLAG-CaSR-Gln27Glu |  | Full-length *CASR* with an N-terminal FLAG tag with the Gln27Glu variant |  | This manuscript |
| pCMV-FLAG-CaSR-Pro221Leu |  | Full-length *CASR* with an N-terminal FLAG tag with the Pro221Leu variant |  | This manuscript |
| pCMV-FLAG-CaSR-Thr828Asn |  | Full-length *CASR* with an N-terminal FLAG tag with the Thr828Asn variant |  | This manuscript |

All sequences are human.

# Figure S1 Growth chart for the proband with the CaSR-Leu723Arg variant


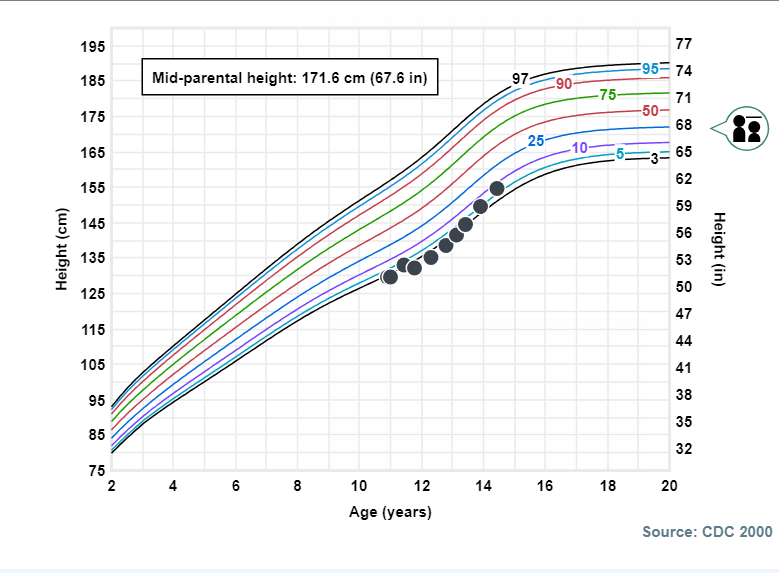


All points shown were measured on a Harpenden Stadiometer in the endocrine clinic.

# Figure S2 Predicted effect of the Arg723 CaSR variant on binding of Gαi1, Gα13, Gαs

**
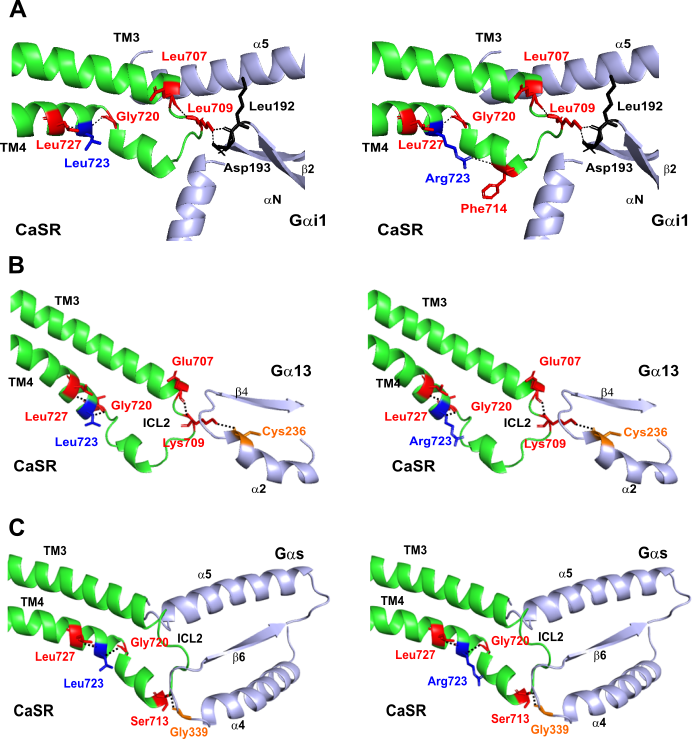
**

(**A**) AlphaFold2 model of the CaSR-Gαi1 interface. Arg723 forms an additional contact with Phe714 of the CaSR which may affect G protein activation. (**B**) AlphaFold2 model of the CaSR-Gα13 interface comprising CaSR ICL2 and the α2-β4 loop of Gα13. (**C**) AlphaFold2 model of the CaSR-Gαs interface comprising CaSR ICL2 and the α4-β6 loop of the G protein. Effects of Arg723 on G13 and Gs coupling are difficult to predict but it is possible that the longer side chain may generate steric hindrance between TM4 and ICL2.

# Figure S3 The CaSR Ala723 variant does not affect activation of Gαi1 or Gαs

**
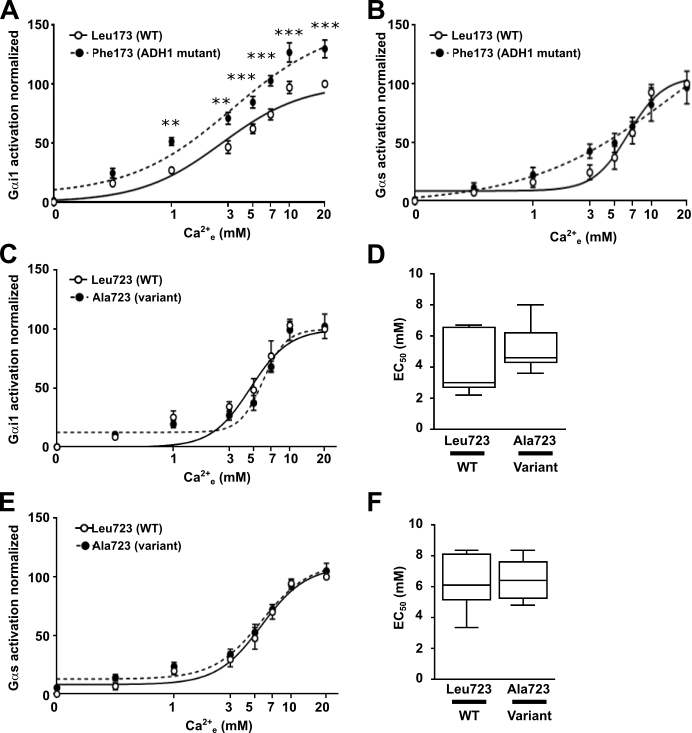
**

Dose-response generated from the AUC of the BRET responses in AdHEK transfected with the CaSR wild-type (WT) or the ADH1-associated Phe173 mutant with (**A**) Gαi1 and (**B**) Gαs. (**C**) Dose-response generated from the AUC of the BRET responses in AdHEK transfected with Gαi1 and CaSR wild-type (WT) or the non ADH1-associated Ala723 variant, with (**D**) EC_50_ values with maximal and minimal values showed by error bars. N=7 biological replicates. (**E**) Dose-response generated from the AUC of the BRET responses in AdHEK transfected with Gαs and CaSR wild-type (WT) or the Ala723 variant, with (**F**) EC_50_ values with maximal and minimal values showed by error bars. N=7 biological replicates. Statistical analyses were performed by two-way ANOVA with Sidak’s test in A, B, C and E, and F-test in D and F.

**Figure S4 Other ADH1 mutations enhance signaling by multiple G proteins**


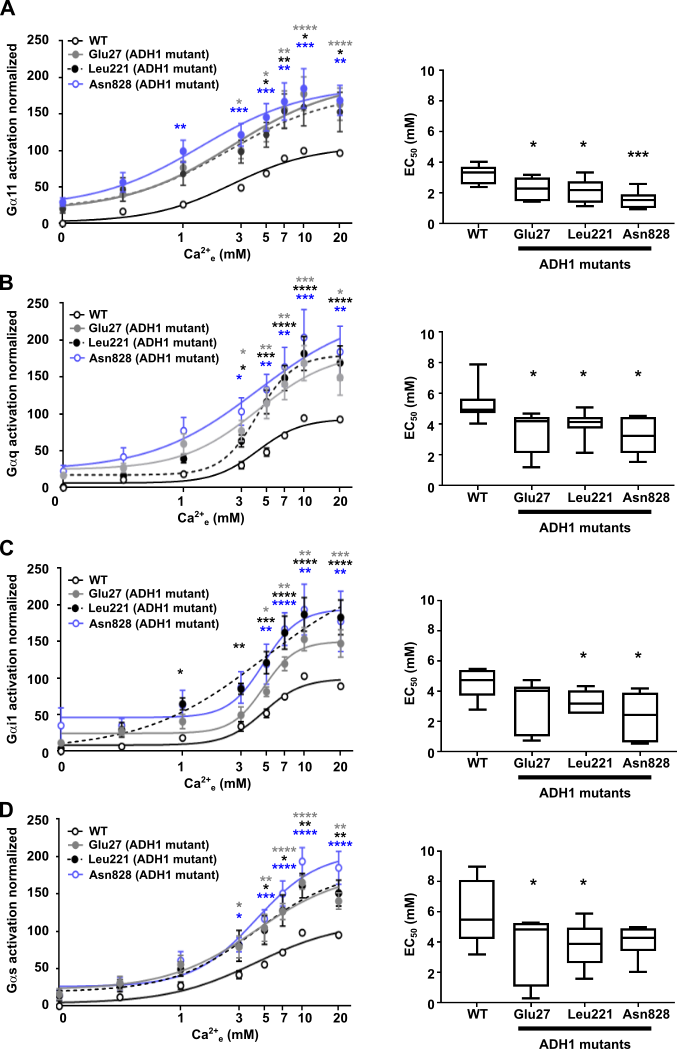


Dose-response generated from the AUC of BRET responses in AdHEK transfected with the CaSR wild-type (WT) or three previously reported ADH1-associated mutants (Gln27Glu, Pro221Leu, Thr828Asn) with (**A**) Gα11, (**B**) Gαq, (**C**) Gαi1 and (**D**) Gαs. EC_50_ values with minimal and maximal values are shown to the right. N=7 biological replicates. Statistical analyses were performed by two-way ANOVA with Sidak’s test for dose-responses and by one-way ANOVA with Dunnett’s test for EC_50_.

# Supplementary References

1. Mannstadt M, Harris M, Bravenboer B, et al. Germline mutations affecting Galpha11 in hypoparathyroidism. *N Engl J Med*. Jun 27 2013;368(26):2532-4. doi:10.1056/NEJMc1300278

2. Akad N, Ungureanu M, Tofan A, Vasiliu I, Tarcau O. Pediatric case of autosomal dominant hypocalcaemia type 2 (ADH2) due to GNA11 gene mutation. presented at: 26th European Congress of Endocrinology; 2024; Stockholm, Sweden.

3. Li D, Opas EE, Tuluc F, et al. Autosomal dominant hypoparathyroidism caused by germline mutation in GNA11: phenotypic and molecular characterization. *J Clin Endocrinol Metab*. Sep 2014;99(9):E1774-83. doi:10.1210/jc.2014-1029

4. Gorvin CM, Stokes VJ, Boon H, et al. Activating Mutations of the G-protein Subunit alpha 11 Interdomain Interface Cause Autosomal Dominant Hypocalcemia Type 2. *J Clin Endocrinol Metab*. Mar 1 2020;105(3):952-63. doi:10.1210/clinem/dgz251

5. Nesbit MA, Hannan FM, Howles SA, et al. Mutations affecting G-protein subunit alpha11 in hypercalcemia and hypocalcemia. *N Engl J Med*. Jun 27 2013;368(26):2476-2486. doi:10.1056/NEJMoa1300253

6. Kwon EJ, Kim MS, Noh ES, et al. The Youngest Infant to Be Diagnosed with Autosomal Dominant Hypocalcemia Type 2 Harboring a Novel Variant of GNA11: A Case Study and Literature Review. *Ann Clin Lab Sci*. May 2022;52(3):494-498.

7. Piret SE, Gorvin CM, Pagnamenta AT, et al. Identification of a G-Protein Subunit-alpha11 Gain-of-Function Mutation, Val340Met, in a Family With Autosomal Dominant Hypocalcemia Type 2 (ADH2). *J Bone Miner Res*. Jun 2016;31(6):1207-14. doi:10.1002/jbmr.2797

8. Tenhola S, Voutilainen R, Reyes M, Toiviainen-Salo S, Juppner H, Makitie O. Impaired growth and intracranial calcifications in autosomal dominant hypocalcemia caused by a GNA11 mutation. *Eur J Endocrinol*. Sep 2016;175(3):211-8. doi:10.1530/EJE-16-0109

9. Takahashi S, Fuchigami T, Suzuki J, Morioka I. A pediatric case of autosomal dominant hypocalcemia type 2. *J Pediatr Endocrinol Metab*. Oct 26 2023;36(10):974-977. doi:10.1515/jpem-2023-0097

10. Chen X, Wang L, Cui Q, et al. Structural insights into the activation of human calcium-sensing receptor. *Elife*. Sep 1 2021;10doi:10.7554/eLife.68578

11. Park J, Zuo H, Frangaj A, et al. Symmetric activation and modulation of the human calcium-sensing receptor. *Proc Natl Acad Sci U S A*. Dec 21 2021;118(51)doi:10.1073/pnas.2115849118

12. Gao Y, Robertson MJ, Rahman SN, et al. Asymmetric activation of the calcium-sensing receptor homodimer. *Nature*. Jul 2021;595(7867):455-459. doi:10.1038/s41586-021-03691-0

13. He F, Wu CG, Gao Y, et al. Allosteric modulation and G-protein selectivity of the Ca(2+)-sensing receptor. *Nature*. Feb 2024;626(8001):1141-1148. doi:10.1038/s41586-024-07055-2

14. Abid HA, Inoue A, Gorvin CM. Heterogeneity of G protein activation by the calcium-sensing receptor. *J Mol Endocrinol*. Jun 21 2021;67(2):41-53. doi:10.1530/JME-21-0058

15. Masuho I, Kise R, Gainza P, et al. Rules and mechanisms governing G protein coupling selectivity of GPCRs. *Cell Rep*. Oct 31 2023;42(10):113173. doi:10.1016/j.celrep.2023.113173

16. Masuho I, Martemyanov KA, Lambert NA. Monitoring G Protein Activation in Cells with BRET. *Methods Mol Biol*. 2015;1335:107-13. doi:10.1007/978-1-4939-2914-6_8

17. Inoue A, Raimondi F, Kadji FMN, et al. Illuminating G-Protein-Coupling Selectivity of GPCRs. *Cell*. Jun 13 2019;177(7):1933-1947 e25. doi:10.1016/j.cell.2019.04.044
